# Docetaxel (Taxotere), cisplatin, UFT, and leucovorin combination chemotherapy in advanced gastric cancer

**DOI:** 10.1038/sj.bjc.6602446

**Published:** 2005-02-22

**Authors:** S C Oh, K H Park, I K Choi, S Y Yoon, S J Kim, J H Seo, C W Choi, B S Kim, S W Shin, J S Kim, Y H Kim

**Affiliations:** 1 Department of Internal Medicine, Division of Hematology/Oncology, Korea University Medical Center, Anam Hospital, 126-1, 5-Ga Anam-Dong, Sungbuk-Gu, Seoul 136-705, Korea

**Keywords:** docetaxel, cisplatin, UFT, stomach neoplasms

## Abstract

We conducted this study to ascertain the efficacy and toxicity of docetaxel and cisplatin combined with oral UFT and leucovorin as a first-line treatment for patients with advanced gastric cancer. In all, 52 patients received courses of docetaxel 60 mg m^−2^ intravenously (i.v.) for 1 h and then cisplatin 75 mg m^−2^ i.v. for 2 h on day 1. Oral UFT at 400–600 mg day^−1^, as determined by body surface area, and leucovorin at 75 mg day^−1^ were administered for 21 consecutive days from day 1, and this was followed by a 7-day drug-free interval. A total of 225 courses were administered, and the median number of courses per patient was four. Four complete responses (7.7%) and 22 partial responses (42.3%) were achieved, giving an overall response rate of 50% (95% Confidence Interval: 36.4–63.6%). The major toxicity was neutropenia, which reached grade 3/4 in 36 patients (69.3%). Grade 3/4 nausea and vomiting was observed in 12 patients (23.1%). Median time to progression was 22 weeks (4 to 156+ weeks), median survival duration was 48 weeks (4 to 156+ weeks), and median response duration was 24 weeks (6–152 weeks). We conclude that docetaxel, cisplatin, oral UFT, and leucovorin combination chemotherapy is effective and tolerable for the treatment of advanced gastric cancer.

Gastric cancer is one of the most common malignancies in Korea (Bae *et al*, 2002) and the second most common cause of cancer-related death worldwide (Boring *et al*, 1994). Despite a decreasing incidence of gastric cancer in the West, the incidence of adenocarcinoma in cardia and at the gastro-oesophageal junction has increased, and 5-year survival for advanced gastric cancer remains poor (Wilke *et al*, 1990). As many patients continue to present with an advanced stage (Karpeh *et al*, 2001), systemic chemotherapy remains the main treatment modality for this disease. Several drugs have shown significant activity against gastric cancer as single agents. These include doxorubicin, cisplatin, and 5-fluorouracil (5-FU), which produce response rates between 19 and 86% (Preusser *et al*, 1988; Findlay and Cunningham, 1993). Combination regimens of these drugs have also shown response rates of 30–50% in phase II studies (Preusser *et al*, 1988; Kim *et al*, 1993). At present, the 5-FU/cisplatin (FP) combination is the most widely used regimen against advanced gastric cancer. Phase II studies of the 5-FP combination reported a 40–51% response rate in previously untreated patients (Kim *et al*, 1993; Rougier *et al*, 1994). FP regimen also produced a significantly higher response rate and a longer time to progression than the FAM (5-FU/doxorubicin/mitomycin) or 5-FU regimens. However, these high response rates did not translate into survival benefits (Kim *et al*, 1993).

To augment the efficacy of the FP regimen, new chemotherapeutic agents have been added, such as taxane, CPT-11, and oxaliplatin. In our previous study, the paclitaxel and 5-FP combination produced a 51% objective response, which suggests that taxane is efficacious in gastric cancer (Kim *et al*, 1999). Docetaxel, a semi-synthetic analogue of paclitaxel, was reported to offer superior response rates and a longer time to progression when added to the FP regimen (5-FU/cisplatin) than FP alone in a randomised phase III trial (Ajani *et al*, 2003). It was also reported that the long-term oral administration of UFT could be as effective as intravenous (i.v.) 5-FU in patients with colorectal cancer (Carmichael *et al*, 2002, Douillard *et al*, 2002). This is supported by our previous experience, namely, that oral UFT/leucovorin was effective in advanced gastric cancer with a response rate of 27%, and tolerable toxicity (Kim *et al*, 1997). Based on these results, we designed a phase II study to evaluate the efficacy of a combined docetaxel-modified FP regimen for the treatment of advanced gastric cancer. To modify the FP regimen, we used oral UFT instead of a 5-FU i.v. infusion and added leucovorin to moderate the antitumour effect of UFT.

## PATIENTS AND METHODS

### Patients

Between September 2001 and September 2003, 52 patients with histologically proven gastric adenocarcinoma, and locally unresectable, recurrent, or metastatic disease were enrolled at the Korea University Medical Center (KUMC). Patients with measurable disease, a performance status ⩽2, and a life expectancy of at least 8 weeks were accepted. Laboratory acceptance parameters included a white blood cell count of >4.0 × 10^3^ l^−1^, an absolute neutrophil count of >1.5 × 10^3^ l^−1^, platelets >100 × 10^3^ l^−1^, serum transaminase <3 × the upper normal limit (UNL), and bilirubin and creatinine (Cr) values of <1.5 × UNL. Contraindications to entry included an active infectious process, an active heart disease, central nervous system involvement, or any concomitant second primary cancer. Patients who had received previous chemotherapy, except adjuvant chemotherapy, were also excluded. Written informed consent was obtained from all patient study subjects, and the study protocol was approved by the institutional review board of KUMC.

### Treatment

On day 1, docetaxel at a dose of 60 mg m^−2^ was administered by i.v. infusion over 1 h. After i.v. hydration for 2 h to prevent nephrotoxicity, cisplatin was administered at 75 mg m^−2^ i.v. over 2 h. These administrations were repeated every 4 weeks. Oral UFT and leucovorin were administered for 21 consecutive days from day 1 to day 21, and this was followed by a 7-day treatment-free period. The total daily dose of UFT (determined by tegafur dosage) was set at three levels according to body surface area (>1.75 m^2^: 600 mg day^−1^; between 1.75 and 1.25 m^2^: 500 mg day^−1^; <1.25 m^2^: 400 mg day^−1^) and it was divided into three doses, which were administered orally every 8 h. It should be noted that UFT was supplied in capsule form, and contained 100 mg of tegafur and 225 mg per capsule. The total daily dose of oral leucovorin (75 mg) was divided into three doses, and these were administered every 8 h regardless of body surface area.

### Dose modification for adverse events

Toxicity was evaluated before each treatment cycle as described in the National Cancer Institute Common Toxicity Criteria (NCI CTC) version 3.0. In cases of grade 3/4 neutropenia of over 5 days duration, or febrile neutropenia, or a platelet count of <50 × 10^3^ l^−1^, docetaxel and cisplatin dosages were reduced by 25%. In cases of grade 3/4 gastrointestinal toxicity, the UFT dosage was decremented by 100  mg day^−1^. UFT and leucovorin were withheld in cases of severe nonhaematologic toxicity, and restarted after symptom resolution. When severe toxicity was noticed at the time of a scheduled administration, treatment was postponed for 1 week until the toxicity resolved. If drug administration was delayed for more than 2 weeks, the subject was excluded from the study. This schedule was repeated 4 weekly until tumour progression or until treatment intolerance developed.

In addition to the above, dolasetron (100 mg) was administered before cisplatin infusion, and lorazepam (0.5 mg) was administered twice on day 1 to prevent delayed emesis, and oral metoclopropamide was prescribed during UFT and leucovorin administration.

### Evaluation

All patients were examined clinically prior to enrollment, and received laboratory evaluations which included: a complete blood cell (CBC) count, electrolytes, blood urea nitrogen (BUN), Cr, liver function test (LFT), and carcinoembronic antigen (CEA). Upper gastrointestinal endoscopy, abdominal computed tomography, and other appropriate procedures were also performed. CBC counts were carried out on days 7, 14, and 28 of every cycle. Chest radiography and laboratory tests including LFT, BUN, Cr, electrolytes, and CEA were performed on day 28 of every cycle. An examination of measurable parameters, such as abdominal computed tomography or chest computed tomography, was used to evaluate treatment response after every two courses of treatment. The World Health Organization (WHO) criteria were adopted (Miller *et al*, 1981) to measure response, which was assessed by the investigators.

### Statistical analysis

All enrolled patients were included in the intention-to-treat analysis of regimen efficacy. The trial was conducted using response rate as the primary end point, and a response rate of 40–50% was anticipated for this regimen as a first-line chemotherapy for advanced gastric cancer. A response rate of 30% was considered as the minimum level of interest in this patient population. Given a true response rate of 50% among 50 patients, the present study provided a 99.67% chance of our being able to determine that the actual response rate exceeded 30%. Time to progression, overall survival, and response duration were secondary end points in Kaplan–Meier analysis. Response duration was defined as the interval between response onset until evidence of progression. Time to progression and overall survival were calculated from the date of study commencement until progression or until the date of the last follow-up or death, respectively.

## RESULTS

### Patients characteristics

Median patient age was 56 years, with a range of 19–77 years (Table 1). In all, 10 patients had a past history of surgery (curative intent in eight and palliative intent in two). One patient had received postoperative adjuvant chemotherapy and one patient intraperitoneal chemotherapy. However, more than 6 months had lapsed between this prior chemotherapy and enrollment into the present study. In total, 42 patients were treatment naive. Median follow-up duration was 11.1 months, and 93, 92.8, and 90.6% of the intended doses of docetaxel, cisplatin, and oral UFT/leucovorin were delivered, respectively.

### Response to chemotherapy

**Figure 1:**
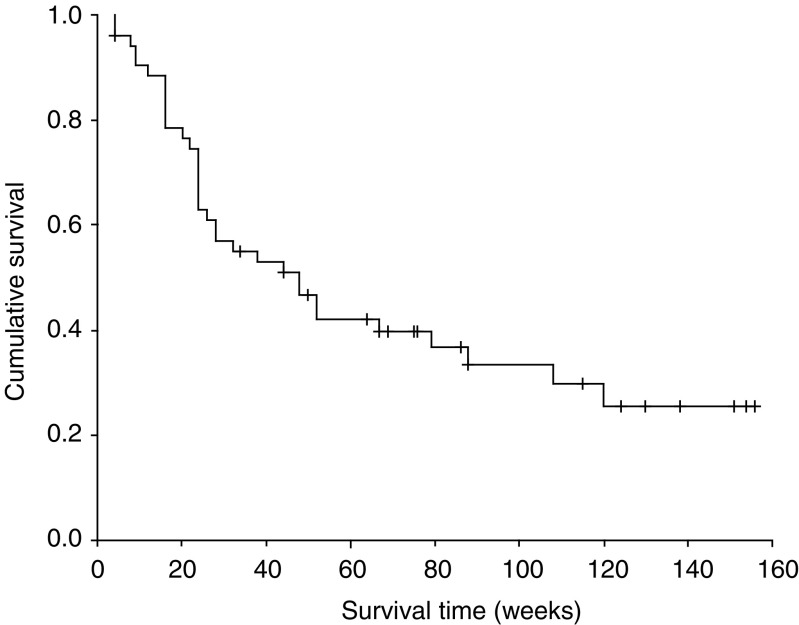
Patient (*N*=52) survival duration.

The 52 enrolled patients received 225 courses of chemotherapy with a median number of courses per patient of four, and a range of 1–8 (Table 2). Five patients who could not complete the second cycle of treatment were not evaluable, three patients were lost at follow-up, and two patients were lost due to treatment-related mortalities (one with sepsis and acute renal failure after the first cycle of treatment and the other patient with grade 4 hepatitis and grade 4 diarrhoea after the second cycle of treatment). Four patients achieved complete response (7.7%) and 22 patients partial response (42.3%). Thus, the overall objective response rate was 50.0% (95% confidence interval (CI), 36.4–63.6%) with a median response duration of 24 weeks (95% CI: 19–29 weeks; range: 6–152 weeks). In all, 17 patients had stable disease and four patients showed disease progression. No complete response was observed among 18 patients with liver or lung metastasis, but four cases of complete response were observed among 34 patients with lymphatic metastasis. No significant difference was observed between partial response and stable disease rates with respect to disease extent. Median time to progression was 22 weeks (95% CI: 16–28 weeks; range: 4 to 156+ weeks), and median survival duration was 48 weeks (95% CI: 25–70 weeks; range: 4 to 156+ weeks) (Figure 1). Four patients with complete response had measurable lymph-node involvement, and surgery was attempted in three of these patients. Although two of the three achieved a pathological complete response, complete resection failed in the other patient, who subsequently developed lung metastasis.

### Toxicity

The main toxicities encountered using this docetaxel/cisplatin/UFT/leucovorin combinatorial therapy were neutropenia and nausea and vomiting (Table 3). Grade 3 or 4 neutropenia occurred in 16 (30.8%) and 20 (38.5%) patients, respectively, and grade 3 or 4 thrombocytopenia was observed in two (3.8%) and one patient (1.9%), respectively. A total of 18 patients required docetaxel and cisplatin dose modification. Of these, the most common cause of dose reduction was prolonged neutropenia or neutropenic fever in 16, hepatitis in one, and early diarrhoea (earlier than day 8) in one. In all, 18 patients required a UFT dose reduction. The most common causes of these dose reductions were: grade 3 or 4 nausea or vomiting in 10, followed by grade 3 or 4 diarrhoea in three. Four patients had multiple toxicities, and required UFT dose modification (one patient had grade 4 mucositis with grade 3 diarrhoea, one had grade 4 mucositis with grade 4 diarrhoea, one had grade 3 mucositis and grade 3 diarrhoea, and the fourth had grade 3 diarrhoea and grade 3 nausea). Grade 2 hepatotoxicity was observed in one patient who remained on a modified UFT dose from the second cycle of treatment. There were three treatment-related mortalities: one patient with grade 3 nausea and grade 3 diarrhoea during the first treatment cycle was lost to follow-up and did not proceed to the next cycle of treatment, another patient died during the first cycle due to severe diarrhoea with grade 4 hepatitis, and the third succumbed to sepsis and acute renal failure. In all, 10 patients required a dose modification of all three anticancer drugs due to multiple adverse effects. Thus, of the 225 courses administered, 56 courses involved a 25% dosage reduction, and seven courses involved more than a 25% dosage reduction.

## DISCUSSION

Docetaxel, a semisynthetic analogue of paclitaxel, continues to show promise as an antigastric cancer agent (Sulkes *et al*, 1994, Einzig *et al*, 1996). Several phase II studies in Europe and USA have reported fairly consistent objective response rates for docetaxel (100 mg m^−2^; 3 weekly) ranging from 17 to 24%. Two Japanese studies adopted 60 mg m^−2^, 3 weekly of docetaxel for the second-line treatment of advanced gastric cancer and achieved response rates of 20 and 22% (Taguchi *et al*, 1998; Mai *et al*, 1999). Moreover, in a study of Korean advanced gastric cancer patients, a 15.9% response rate was obtained with single-agent docetaxel at 75 mg m^−2^ every 3 weeks (Bang *et al*, 2002). Synergism between docetaxel and cisplatin was suggested by the Swiss Group for Clinical Cancer Research (SAKK) and by the European Institute for Oncology (EIO) in Milan (Roth *et al*, 2000; Kettner *et al*, 2001). These two reports found that the docetaxel (85 mg m^−2^) and cisplatin (75 mg m^−2^) combination produced a response rate of 52% in 48 patients, a median time to progression of 6.6 months, and a median overall survival time of 9 months by intention-to-treat analysis. In addition, although therapeutic efficacy was not the primary objective, a phase I study of weekly docetaxel, and 24-h infusion of high-dose fluorouracil and leucovorin, and cisplatin produced a response in 16 of 26 (61.5%, two complete responses and 14 partial responses) evaluable advanced gastric cancer patients (Chen *et al*, 2002).

To augment the therapeutic effect of docetaxel/cisplatin, a randomised phase II study compared the combination docetaxel (75 mg m^−2^), cisplatin (75 mg m^−2^), and 5-FU (750 mg m^−2^) with docetaxel (85 mg m^−2^) and cisplatin (75 mg m^−2^) (Roth and Ajani, 2003). The study showed that the addition of 5-FU increased the response rate (44% *vs* 30%). Another randomised phase II study compared a combination of docetaxel and cisplatin (DC) with docetaxel, cisplatin, and 5-FU (DCF). By intention-to-treat analysis, the response rate of DC was 28% and that of DCF was 43%. This higher response rate achieved by DCF led to a comparative phase III clinical trial against cisplatin plus 5-FU (CF) (Roth and Ajani, 2003). In an interim analysis, DCF was found to have an extended time to progression (5.2 *vs* 3.7 months) and an increased response rate (39 *vs* 23%) (Ajani *et al*, 2003). Thus, the docetaxel/cisplatin/5-FU combination may emerge as the standard regimen for the treatment of advanced gastric cancer.

In our study, oral UFT was used instead of i.v. 5-FU. Oral UFT is known to generate plasma 5-FU levels that are similar to those of i.v. 5-FU, and oral UFT can produce up to 10-fold higher 5-FU concentrations in tumour tissue than in normal tissue. Furthermore, oral UFT has several advantages over i.v. 5-FU in terms of its clinical application. First, UFT can be administered enterally, which promotes compliance and maintains a constant plasma level of 5-FU. Second, it can be discontinued whenever severe toxicity develops, which prevents further toxicity-associated deterioration. Moreover, after toxicity subsidence, patients can restart the schedule. During the past decade, it has been recognised that leucovorin modulates 5-FU and enhances antitumour efficacy (Buroker *et al*, 1994), and in our previous study, oral UFT/leucovorin was found to have a role in the palliative chemotherapy of advanced gastric cancer (Kim *et al*, 1997).

In the present study, neutropenia was the main haematological toxicity, and grade 3 or 4 neutropenia was observed in 16 (30.8%) and 20 (38.5%) patients, respectively. Febrile neutropenia developed in seven patients, and of these six recovered with support from granulocyte colony-stimulating factor, and the dose was subsequently reduced. The remaining patient did not receive a subsequent course due to a sepsis-related mortality. Severe neutropenia was also observed in a SAKK and EIO study (Roth *et al*, 2000), and another study also reported neutropenia as the major dose-limiting toxicity; 45% showed grade 3–4 neutropenia, and among these, two episodes of febrile neutropenia occurred (Chen *et al*, 2002). In the present study, nonhaematological toxicity was moderate, and grade 3 nausea and vomiting developed in 12 (20%) patients, and grade 3/4 diarrhoea or mucositis developed in less than 10% of patients. These nonhaematologic toxicities were successfully managed by symptomatic treatment. Nine patients developed grade 3 or 4 diarrhoea during oral UFT and leucovorin treatment, which necessitated oral UFT and leucovorin treatment cessation. However, after symptomatic relief, treatment was restarted in seven of the nine. Of the two remaining patients, one died after the first treatment cycle and one patient was lost to follow-up. The following course of treatment was restarted in the seven with a dose reduction of UFT at the minus one level (a decrement of 100  mg day^−1^). These results suggest that the regimen containing oral UFT has a better toxicity profile than DCF (Ajani *et al*, 2003), and support previous findings of studies that reported that the long-term oral administration of UFT might be as useful as i.v. 5-FU in patients with colorectal cancer (Carmichael *et al*, 2002, Douillard *et al*, 2002).

The present study demonstrates that our regimen is highly effective and has a feasible toxicity profile against advanced gastric cancer with a 50.0 % overall response rate and a 48-week median survival. Owing to the 1-day infusion schedule, this regimen can be administered on an outpatient basis without disrupting daily life. We conclude that the oral UFT/leucovorin/docetaxel/cisplatin combination is likely to be active against advanced gastric cancer. Nevertheless, a randomised phase III trial is warranted to compare this regimen with docetaxel/cisplatin/i.v. 5-FU.

## Figures and Tables

**Table 1:** Patient characteristics

Total no. of patients	52
No. of nonevaluable patients	5
Median age in year	56
Range	19–77
Male/female	37/15
*ECOG performance status*	
0	4
1	39
2	9
	
*Site of metastasis*	
Lymph node	34
Liver	15
Lung	3
	
*Previous treatment*	
None	42
Operation	10
Palliative/curative	2/8
Chemotherapy	2
IP/adjuvant	1/1

ECOG=Eastern cooperative oncology group.

**Table 2:** Treatment response shown by gastric cancer patients

Total no. of chemotherapy cycle	225
Median	4
Range	1–8
*Dose delivery*	
Docetaxel	93.0%
Cisplatin	92.8%
UFT/leucovorin	90.6%
	
*Response* (%) (*n*=*52*)	
CR	4 (7.7%)
PR	22 (42.3%)
SD	17 (32.7%)
PD	4 (7.7%)
NE	5 (9.6%)
Overall response (95% CI)	50.0% (36.4–63.6%)
Median time to progression, week, range	22 (4 to 156+)
Median survival duration, week, range	48 (4 to 156+)
Median response duration, week, range	24 (6–152)

CR=complete response; PR=partial response; SD=stable disease; PD=progressive disease;

NE=not evaluable.

**Table 3:** Major toxicities – NCI CTC version 2.0 (*N*=52)

	**Number of patients (%)**		
**Toxicity**	**Grade I/II**	**III**	**IV**
Neutropenia	5 (9.6)	16 (30.8)	20 (38.5)
Thrombocytopenia	2 (3.8)	2 (3.8)	1 (1.9)
Anaemia	10 (19.2)	0	1 (1.9)
Mucositis	3 (5.8)	1 (1.9)	2 (3.8)
Diarrhoea	5 (9.6)	3 (5.8)	6 (11.5)
Nausea/vomiting	7 (13.5)	12 (23.1)	0
Hepatotoxicity	1 (1.9)	0	1 (1.9)
Alopecia	27 (45)	0	0

NCI CTC=National Cancer Institute Common Toxicity Criteria.
